# Cathelicidin preserves intestinal barrier function in polymicrobial sepsis

**DOI:** 10.1186/s13054-020-2754-5

**Published:** 2020-02-10

**Authors:** Jeffery Ho, Hung Chan, Yonghao Liang, Xiaodong Liu, Lin Zhang, Qing Li, Yuchen Zhang, Judeng Zeng, Felix N. Ugwu, Idy H. T. Ho, Wei Hu, Johnny C. W. Yau, Sunny H. Wong, Wai Tat Wong, Lowell Ling, Chi H. Cho, Richard L. Gallo, Tony Gin, Gary Tse, Jun Yu, Matthew T. V. Chan, Czarina C. H. Leung, William K. K. Wu

**Affiliations:** 1 Department of Anaesthesia and Intensive Care and Peter Hung Pain Research Institute, The Chinese University of Hong Kong, Shatin, Hong Kong Special Administrative Region China; 2 State Key Laboratory of Digestive Diseases, Li Ka Shing Institute of Health Sciences, and Centre for Gut Microbiota Research, The Chinese University of Hong Kong, Shatin, Hong Kong Special Administrative Region China; 3 Department of Medicine and Therapeutics, The Chinese University of Hong Kong, Hong Kong Special Administrative Region, Shatin China; 4 Laboratory of Molecular Pharmacology, School of Pharmacy, Southwest Medical University, Luzhou, Sichuan China; 5 Department of Dermatology, The University of California, San Diego, USA

**Keywords:** LL-37, Sepsis, Bacterial translocation, Antimicrobial peptide

## Abstract

**Objectives:**

The intestinal epithelium compartmentalizes the sterile bloodstream and the commensal bacteria in the gut. Accumulating evidence suggests that this barrier is impaired in sepsis, aggravating systemic inflammation. Previous studies reported that cathelicidin is differentially expressed in various tissues in sepsis. However, its role in sepsis-induced intestinal barrier dysfunction has not been investigated.

**Design:**

To examine the role of cathelicidin in polymicrobial sepsis, cathelicidin wild-(*Cnlp*^+/+^) and knockout (*Cnlp*^−/−^) mice underwent cecal-ligation and puncture (CLP) followed by the assessment of septic mortality and morbidity as well as histological, biochemical, immunological, and transcriptomic analyses in the ileal tissues. We also evaluated the prophylactic and therapeutic efficacies of vitamin D3 (an inducer of endogenous cathelicidin) in the CLP-induced murine polymicrobial sepsis model.

**Results:**

The ileal expression of cathelicidin was increased by three-fold after CLP, peaking at 4 h. Knockout of *Cnlp* significantly increased 7-day mortality and was associated with a higher murine sepsis score. Alcian-blue staining revealed a reduced number of mucin-positive goblet cells, accompanied by reduced mucin expression. Increased number of apoptotic cells and cleavage of caspase-3 were observed. *Cnlp* deletion increased intestinal permeability to 4kD fluorescein-labeled dextran and reduced the expression of tight junction proteins claudin-1 and occludin. Notably, circulating bacterial DNA load increased more than two-fold. Transcriptome analysis revealed upregulation of cytokine/inflammatory pathway. Depletion of *Cnlp* induced more M1 macrophages and neutrophils compared with the wild-type mice after CLP. Mice pre-treated with cholecalciferol (an inactive form of vitamin D3) or treated with 1alpha, 25-dihydroxyvitamin D3 (an active form of VD3) had decreased 7-day mortality and significantly less severe symptoms. Intriguingly, the administration of cholecalciferol after CLP led to worsened 7-day mortality and the associated symptoms.

**Conclusions:**

Endogenous cathelicidin promotes intestinal barrier integrity accompanied by modulating the infiltration of neutrophils and macrophages in polymicrobial sepsis. Our data suggested that 1alpha, 25-dihydroxyvitamin D3 but not cholecalciferol is a potential therapeutic agent for treating sepsis.

## Introduction

Sepsis is a life-threatening organ dysfunction accompanied by systemic inflammation and immunosuppression as a consequence of the host response to microbial infections. Sepsis which carries high mortality and morbidity in the intensive care unit remains a major health burden [1]. Therefore, there is a compelling need for developing novel sepsis therapies.

The pathogenesis of sepsis has been attributed, at least in part, to the loss of intestinal epithelial barrier. As the first line of defense, the gut intestinal epithelial barrier impedes the translocation of commensal bacteria from the gut lumen into the bloodstream. Accumulating evidence suggests that the intestinal barrier function is impaired during systemic inflammation as in sepsis. These include epithelial apoptosis, disruption of tight junctions leading to an increase in intestinal permeability [2, 3]. The impaired gut barrier function may increase the risk of bacterial translocation from the gut lumen to the bloodstream, aggravating systemic inflammation. Clinically, bacterial translocation from the gut into the bloodstream has been demonstrated in patients with postoperative sepsis [4]. An abnormal and severe derangement of intestinal permeability upon admission to an intensive care unit was found to predict subsequent development of multiple organ failure [5]. However, the underlying mechanism of sepsis-associated gut barrier dysfunction remains elusive.

Cathelicidin represents one of the most important classes of antimicrobial peptides in mammals. It has bactericidal property, inhibits endotoxin-induced pyroptosis of leukocytes, suppresses the release of inflammatory mediators, and protects endothelial cells from apoptosis [6, 7]. Cathelicidin can be induced by vitamin D3 (VD3), which has therapeutic properties outside of its classic functions related to bone and calcium homeostasis [8, 9]. In particular, a growing body of evidence has shown the antibiotic-like properties of vitamin D [10]. Thus, this natural compound may prove to be effective against sepsis, as an adjunct treatment modality. Previously, Chen and his colleagues suggested that VD3 exerts protective effects during infections by upregulating the expression of cathelicidin and beta-defensin 2 in phagocytes and epithelial cells [11]. Another study found that systemic LL-37 (human cathelicidin) levels may be regulated by VD3 status [12]. In our study, we aimed to investigate the role of murine cathelicidin-related antimicrobial peptide (mCRAMP), a rodent antimicrobial peptide analogous to human cathelicidin LL-37, in maintaining gut barrier function in sepsis and to explore the relationship between vitamin D3 status and cathelicidin production in CLP mice model.

## Materials and methods

### Animals

129/SVJ wild-type (*Cnlp*^+/+^) and cathelicidin-knockout (*Cnlp*^−/−^) mice were used. These mouse strains were generated as previously described [13]. All animals were male and 8 to 10 weeks old. They were maintained in the Laboratory Animal Services Center of the Chinese University of Hong Kong at a controlled temperature of 25 °C ± 1 °C, relative humidity 55% ± 5%. A cycle of 12 h light/12 h dark was maintained prior to the experiments.

### Cecal ligation and puncture

Polymicrobial sepsis was induced by cecal-ligation and puncture (CLP) [14]. Under anesthesia with intra-peritoneal injection of ketamine (75 mg/kg) and xylazine (10 mg/kg), a 1-cm midline incision was made on the anterior abdomen. The cecum was exposed and ligated at 50% from the distal end. A through-and-through puncture was performed with a 22-gauge needle to induce sepsis. The cecum was then placed back into the peritoneal cavity. Sham-operated animals underwent abdominal incision and intestinal manipulation with neither ligation nor puncture. All animals were given 1 ml of normal saline by subcutaneous injection and placed on a warm towel immediately after the surgery. The survival rates and septic severity were recorded every 12 h until 7 days after the surgery. No antibiotic was given to the CLP-operated mice in order to assess the systemic inflammation after surgery [15, 16] Concerning animal welfare, buprenorphine (0.01 mg/kg) was administered to the mice after surgery. Mice were given buprenorphine (0.01 mg/kg) daily until the end of the experiment if necessary.

#### *VD3* prophylaxis

VD3 were purchased from Sigma Chemical Co. (St., Louis, MO). In the water control group, mice were pretreated with water by oral gavage at 48 h, 24 h, and 1 h before CLP. In the VD3 prophylaxis group, mice were pretreated with three doses of VD3 (50 μg/kg) by oral gavage at 48 h, 24 h, and 1 h before CLP. The doses of VD3 used in the present study were referred to as others [17] .

#### Treatment with active VD3

1alpha, 25-dihydroxyvitamin D3 (1alpha, 25(OH)_2_VD3) were purchased from Cayman Chemical Co. (Ann Arbor, MI). Mice were treated with water or 1alpha, 25(OH)_2_VD3(50μg/kg) for 7 days after CLP by intraperitoneal injection.

### Assessment of sepsis morbidity

Septic morbidity was evaluated by Murine Sepsis Severity (MSS) score. Briefly, a score was assigned based on appearance, level of consciousness, activity, response to the stimulus, eyes, respiratory rate, and respiration quality.

### Biochemical analyses

Serum alanine transaminase (ALT) and aspartate aminotransferase (AST) levels were determined using Vet Test Chemistry Analyzer (IDEXX) according to the manufacturer’s instructions. Serum vitamin D levels were measured using the vitamin D ELISA kit (#501050, Cayman).

### Reverse transcription-quantitative PCR

Total RNA was extracted from ileal tissues by RNAiso Plus reagent according to the commercial protocol (TaKaRa, Japan). For each specimen, a total of 500 ng RNA was reverse-transcribed into cDNA using PrimeScript RT reagent (TaKaRa, Japan). Quantitative real-time PCR was performed with Quantstudio 12 K Flex Real-time PCR system (Life Technologies, Thermo Fisher Scientific, MA, USA) using primers targeting *Muc1*, *Muc2*, *Muc3*, *Muc4*, *Cnlp*, and *β-actin* [18–21].

### Histology and immunofluorescence

Harvested ileal tissues were washed briefly in cold phosphate-buffered saline and fixed in Carnoy’s solution (60% ethanol, 30% chloroform, and 10% glacial acetic acid) at 4 °C for 4 h. Fixed tissues were stored in 80% ethanol at 4 °C before tissue processing. Processed sections were stained with Alcian-blue followed by periodic acid Schiff reaction. Expression of cathelicidin was detected in a series of ileal specimens harvested in the acute phase of sepsis. For immunofluorescence, dewaxed and rehydrated slides of murine ileal sections were blocked with 10% bovine serum immunofluorescence buffer (0.1% bovine serum albumin, 0.2% Triton X-100, 0.5% TWEEN 20 in phosphate-buffered saline) and then incubated with mouse mCRAMP (Santa Cruz,1:200) antibodies overnight at 4 °C followed by Alexa Fluor anti-mouse 546 secondary antibodies (1:2000). 4′,6-diamidino-2-phenylindole (DAPI) was used for DNA counterstain. Fluorescent images were captured using a confocal microscope (Leica).

### Apoptosis assay

Apoptosis was assessed by an in situ cell death detection kit (Roche Applied Science) and confirmed by immunoblotting using antibodies targeting caspase-3 and cleaved caspase-3.

### Intestinal permeability assay and tight junction proteins

Mice were gavaged with 4 kD fluorescein isothiocyanate (FITC)-dextran (500 mg/kg) at 21 h after CLP or sham surgery. After 3 h, blood was collected and the intensity of FITC determined by fluorometry. The expression of tight junction proteins, claudin-1, and occluding was evaluated by immunoblotting.

### Profiling of ileal transcriptome

Total RNA was extracted from ileal tissues at 24 h after CLP or sham surgery using RNAiso Plus (TaKaRa, Shiga, Japan). The poly-A RNA was purified and used for library construction. The sample libraries were sequenced with the Illumina HiSeq 2000 sequencing system (Illumina, San Diego, CA, USA). Clean reads were aligned to *Mus musculus* primary DNA index files (release-94). Transcripts were then assembled by Cufflinks [22]. Differentially expressed genes (DEGs) between *Cnlp*^+/+^ CLP and *Cnlp*^+/+^ Sham mice, as well as *Cnlp*^−/−^ CLP and *Cnlp*^−/−^ Sham mice were identified using edgeR packages. Short Time-series Expression Miner (STEM) software was adopted for the identification of co-expression gene clusters among four groups of mice. The co-expression pattern of particular gene clusters was confirmed and visualized by Pheatmap R package. Pathway analysis was performed with enrichr R package and visualized by ggplot2. Protein–protein interaction network was generated in STRING. The interaction between genes was defined according to “experiments,” “databases,” and “co-expression.” The network topology was analyzed with the “NetworkAnalyzer” plugin in cystoscope.

### Intestinal epithelial cell isolation

The small intestine was prepared by cutting the gut about 1 cm downstream from the stomach and 1 cm upstream from the cecum. Forceps were used to remove Peyer’s patches and the attached mesenteric fat carefully. The small intestine was then placed into a 50 mL conical tube containing 30 mL of CMF HBSS (Hank’s balanced salt solution with phenol red, Ca2^+^, and Mg2^+^-free) with 5% FBS and 2 mM EDTA and shook at 250 rpm for 20 min at 37 °C in order to remove epithelial cells and intraepithelial lymphocytes. The intestine was rapidly minced and incubated in 20 mL of pre-warmed collagenase solution (1.5 mg/mL of collagenase VIII and 40 μg/mL of DNase I in CMF HBSS/FBS) with a shaking frequency of 200 rpm for 20 min at 37 °C for digestion [23].

### Flow cytometry

After blocking Fc receptors with anti-mouse CD16/CD32 (BD Biosciences), small intestinal epithelial cells were stained with anti-mouse Ly-6G (BioLegend), anti-mouse F4/80 (BD Biosciences), anti-mouse CD86 (BD Biosciences), anti-mouse CD206 (BD Biosciences), and anti-mouse CD45 (BD Biosciences). The stained cells were analyzed on a FACSCalibur flow cytometer (BD Biosciences). The data were analyzed using FlowJo Software (FlowJo, Ashland, OR). Neutrophils were defined as Ly6G^+^ cells and macrophages as F4/80^+^ cells and M1 macrophages as F4/80^+^ CD86^+^ and M2 macrophages as F4/80^+^ CD206^+^. Lymphocytes were defined as CD45^+^ cells.

### Statistical analysis

Multiple group comparisons were performed by two-way ANOVA or non-parametric Kruskal-Wallis followed by the Tukey’s *t* test. Mortality was compared by Kaplan-Meier survival curves and analyzed by the log-rank test. *P* values less than 0.05 were considered statistically significant.

## Results

### Endogenous cathelicidin protects against peritonitis-induced polymicrobial sepsis in mice

**Fig. 1:**
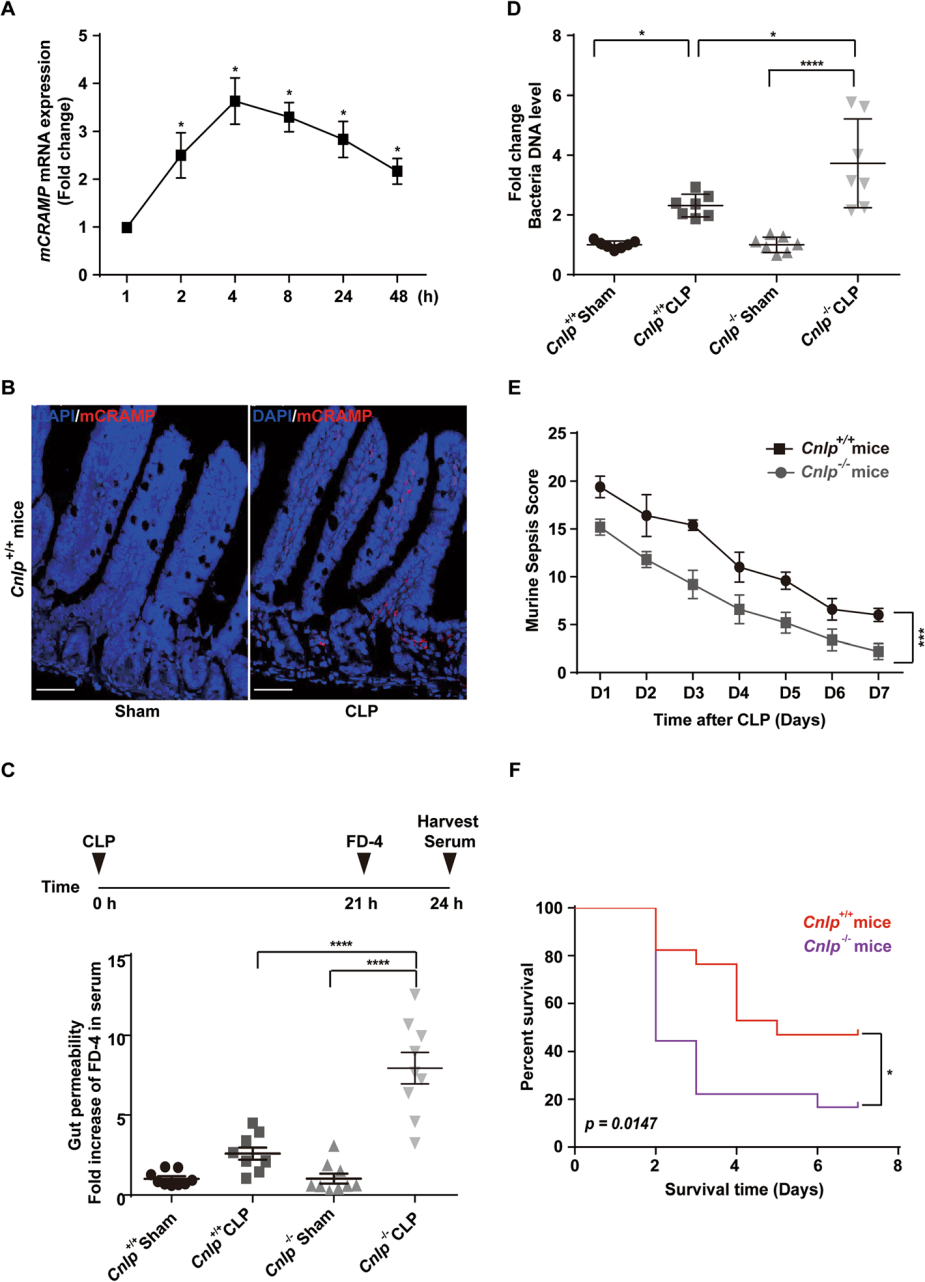
Murine cathelicidin-related antimicrobial peptide (mCRAMP) is upregulated after cecal-ligation and puncture (CLP) induced sepsis in wild-type (WT) mice (*n* = 6 per group) from which total RNA and protein were collected from distal ileum over a period of time for **a** real-time PCR and **b** immunofluorescence for mCRAMP. Genetic knockout (KO) of *Cnlp* led to **f** reduced survival and **e** higher sepsis severity score (*n* = 18 for WT mice; *n* = 17 for KO mice). FITC dextran 4 kD was orally gavaged at 21 h after CLP with serum harvested after 3 h. Genetic KO of *Cnlp* led to **c** increased serum concentration of FITC-labeled dextran 4 kD (FD-4) and **d** increased bacterial DNA upon experimental sepsis. Error bars denote standard error of the mean. *P < 0.05; ***P < 0.001; ****P < 0.0001

Given an increase of mCRAMP mRNA and protein expression in the ileum of *Cnlp*^+/+^ mice following CLP (Fig. 1a, b), we hypothesized that mCRAMP was an important peptide in the pathogenesis of sepsis. To ascertain the significance of this antimicrobial peptide during sepsis, *Cnlp*^−/−^, and wild-type mice were included in this study. All of the mice that underwent sham surgery survived throughout 7 days (data not shown). *Cnlp*^−/−^ mice had increased 7-day mortality (hazard ratio = 2.229, 95% CI 1.491–7.550) (Fig. 1f) and significantly higher MSS score (Fig. 1e) and higher level of fluorescein dextran entering the bloodstream upon CLP when compared with that of *Cnlp*^+/+^ mice (Fig. 1c). To ascertain the association between bacterial load and sepsis morbidity and mortality, total bacterial DNA was determined by quantitative PCR. Compared to wild-type mice at 24 h after CLP, *Cnlp*^−/−^ mice had more than two-fold increase in bacterial DNA in the blood (Fig. 1d).

### Mucin production is reduced in Cnlp^−/−^ mice following CLP-induced sepsis

**Fig. 2:**
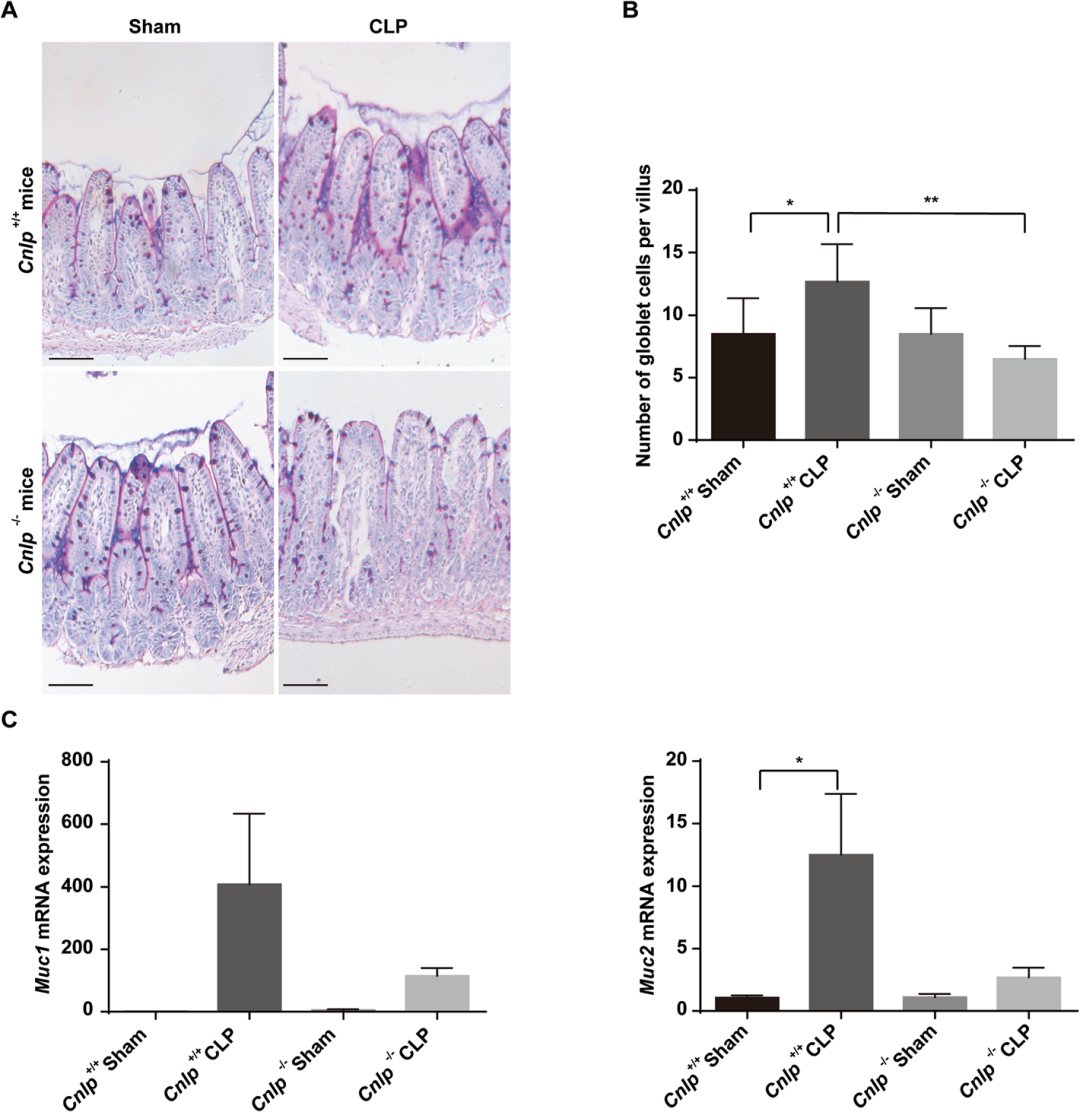
Effects of cecal-ligation and puncture (CLP) or sham surgery (Sham) on acid mucin in distal ileum of cathelicidin wild-type (*Cnlp*^+/+^) or knockout (*Cnlp*^−/−^) mice (*n* = 6 per group) at 24 h after CLP as determined by quantitative real-time PCR and **a** Alcian blue perioidic acid Schiff reaction, respectively. **b** The number of acid-mucin-producing globlet cells per villus and **c** the expression of mucin genes *MUC1* and *MUC2* were compared. Error bars represent standard error of the mean. *P < 0.05; **P < 0.01

Alcian-blue staining demonstrated that the number of goblet cells per villus in the intestine among the knockout group was significantly lower compared with the wild-type mice (Fig. 2a, b). To further investigate the underlying mechanisms, we performed real-time quantitative PCR targeting mucin genes *Muc1* and *Muc2.* Among the *Cnlp*^*−/−*^ mice undergone CLP, the expression levels of *Muc2* (Fig. 2c) were significantly reduced as compared to their wild-type counterparts.

### Tight junctions of intestinal epithelial cells were reduced in septic cathelicidin-knockout mice

**Fig. 3:**
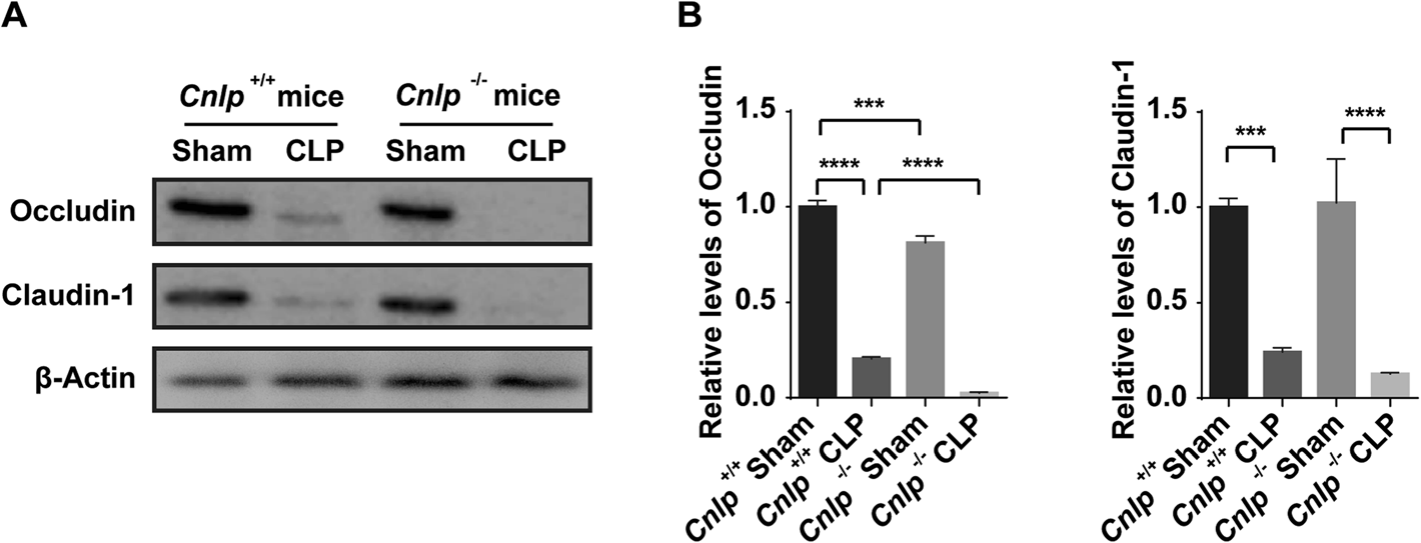
Deletion of *Cnlp* reduced tight junction of intestinal epithelial cells. The protein levels of **a**, **b** occludin and claudin-1 were detected in cathelicidin-knockout (*Cnlp*^−/−^) mice (*n* = 6) compared to wild-type mice (*Cnlp*^+/+^) (*n* = 6) after CLP-induced sepsis by immunoblotting. Error bars denote standard error of the mean. ***P < 0.001; ****P < 0.0001

To ascertain the reasons for higher gut permeability, tight junction proteins, namely, occludin and claudin-1, were determined by immunoblotting. Compared to wild-type mice at 24 h after CLP, *Cnlp*^−/−^ mice had lower expression of occludin and claudin-1 (Fig. 3a, b). Real-time PCR and transcriptome analysis showed a concordant downregulation of these two genes at the mRNA level (data not shown).

### Endogenous cathelicidin protects against intestinal epithelial cell apoptosis in sepsis

**Fig. 4:**
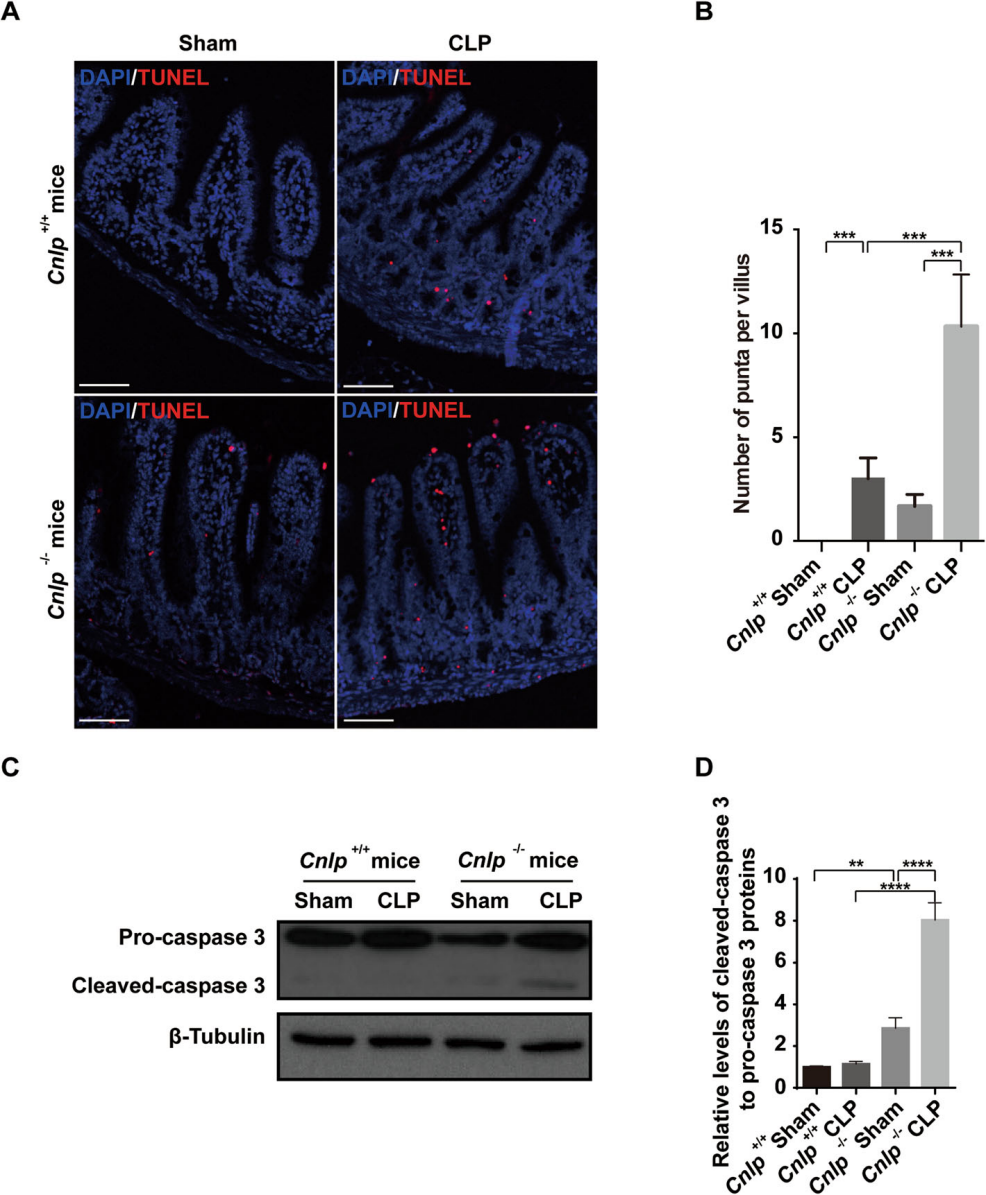
Increased apoptosis was detected in cathelicidin-knockout (*Cnlp*^−/−^) mice (*n* = 6) compared to wild-type mice (*Cnlp*^+/+^) (*n* = 6 per group) after CLP-induced sepsis as demonstrated by **a**, **b** TUNEL staining and **c**, **d** immunoblotting for cleaved caspase-3. All specimens were collected at 24 h after CLP or Sham surgery. Error bars denote standard error of the mean. **P < 0.01; ***P < 0.001; ****P < 0.0001

To determine the extent of apoptosis in distal ileum upon experimental sepsis, TUNEL labeling was used. By 24 h after CLP, the number of TUNEL positive punta per villus increased considerably in both *cnlp*^+/+^ and *cnlp*^−/*−*^ mice (Fig. 4s). The depletion of mCRAMP exaggerated the magnitude of apoptosis in the distal ileum by more than three-fold (Fig. 4b). Consistently, cleavage of caspase-3 was detected in immunoblotting, confirming active apoptosis (Fig. 4c, d).

### Ileal transcriptome identified signaling pathways regulated by cathelicidin

**Fig. 5:**
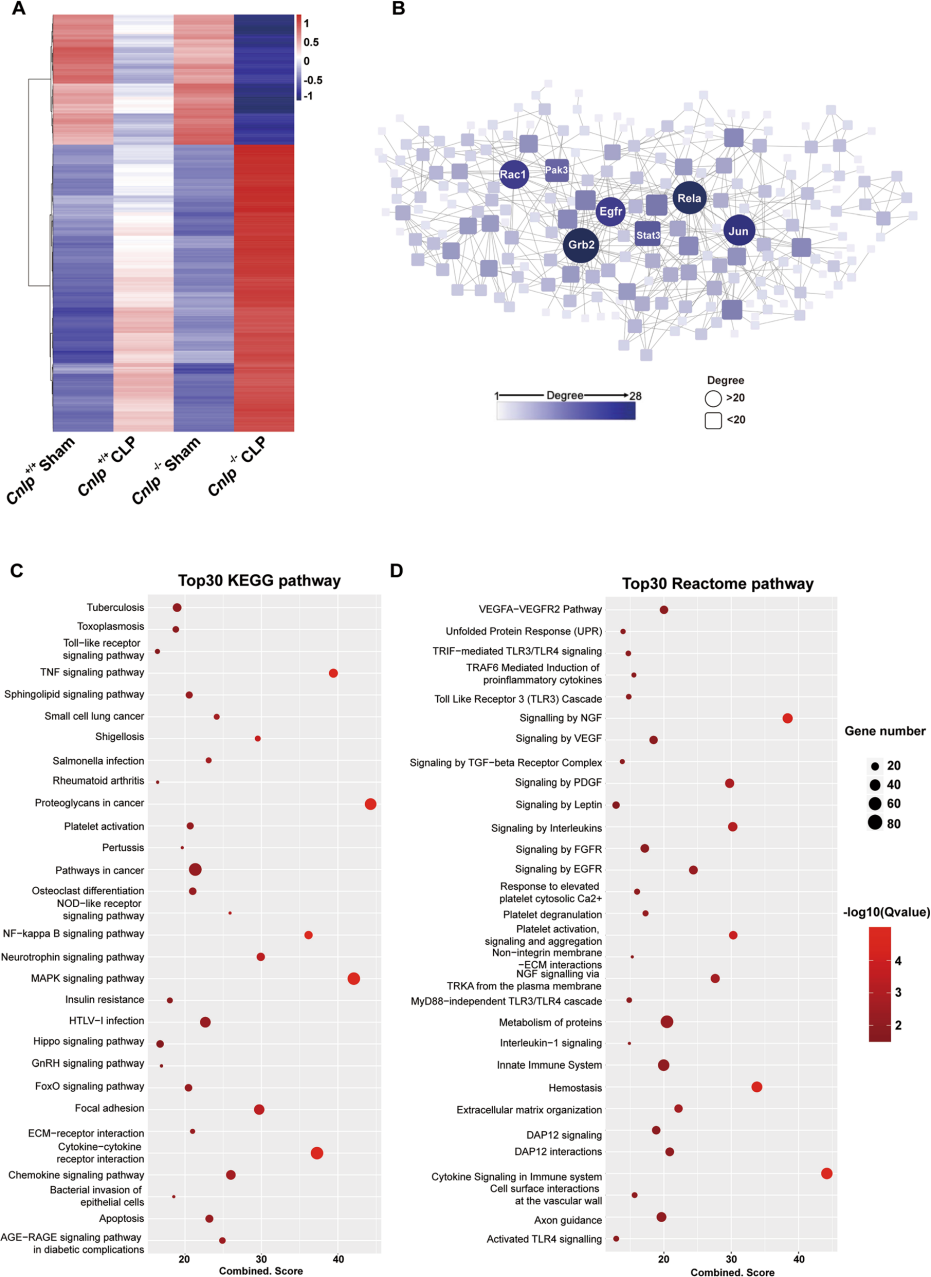
Ileal transcriptomes of septic cathelicidin wild-type and knockout mice. **a** Heatmap was generated using genes in cluster 8 and cluster 16. The transcriptome datasets from the septic and non-septic wild-type mice but not the cathelicidin knockout mice were published in Inflamm Res. 2019; 68 [9]:723–726. **b** Protein-protein interaction network was constructed in STRING using the source of “experiments,” “databases,” and “co-expression” and visualized by cytoscape. Nodes in round shape have a degree large than 22. Several inflammation-related genes were highlighted as hub genes according to the topology analysis. **c**, **d** Top 30 KEGG and Reactome pathways were plotted. A series of inflammation related pathways were enriched by both sources

We performed RNA sequencing to profile the transcriptomes of ileal tissues in the following four groups: *Cnlp*^+/+^ sham, *Cnlp*^+/+^ CLP, *Cnlp*^−/−^ sham, and *Cnlp*^−/−^ CLP at 24 h after surgery. STEM analysis identified a total of 19 significant co-expression gene clusters (Additional file 1: Figure S1), among which 2 co-expression patterns, i.e., cluster8:1-2-1-4 (*Cnlp*^+/+^ Sham-*Cnlp*^+/+^ CLP-*Cnlp*^−/−^ Sham-*Cnlp*^−/−^ CLP) and cluster16:1-0.5-1-0.25 appeared to be best correlated to the differences of MSS scores between groups. In the cluster8, genes were significantly upregulated after CLP compared with sham surgery in wide-type mice (2 vs 1). The fold changes of these genes were further increased (4 vs 1) between CLP and sham surgery in *Cnlp*^−/−^ mice. In an inverse pattern, genes from cluster16 were downregulated by CLP surgery with a greater extent in *Cnlp*^−/−^ mice than in *Cnlp*^+/+^ mice. Heatmap analysis further confirmed the gene expression pattern among groups (Fig. 5a). These genes were most likely to contribute to the severe septic symptoms in *Cnlp*^−/−^ mice compared to wide-type mice. Then protein–protein interaction network was constructed using the genes in cluste8 (Fig. 5b). Topology analysis identified several “hub” genes with a degree of 16 or higher. Interestingly, these hub genes, e.g., *Rac1*, *Pak3*, *Grb2*, *Stat3*, *Rela*, and *Jun*, were all reported to play critical roles in inflammatory signaling (Fig. 5b), implying that dysregulated inflammatory responses might have aggravated the septic phenotype in *Cnlp*^−/−^ mice. Indeed, a series of inflammation-related pathways were enriched in the KEGG (Kyoto Encyclopedia of Genes and Genomes; Fig. 5c) and Reactome (Fig. 5d) pathway analyses.

### Deletion of endogenous cathelicidin increases neutrophils and M1 macrophages in the intestines of septic mice

**Fig. 6:**
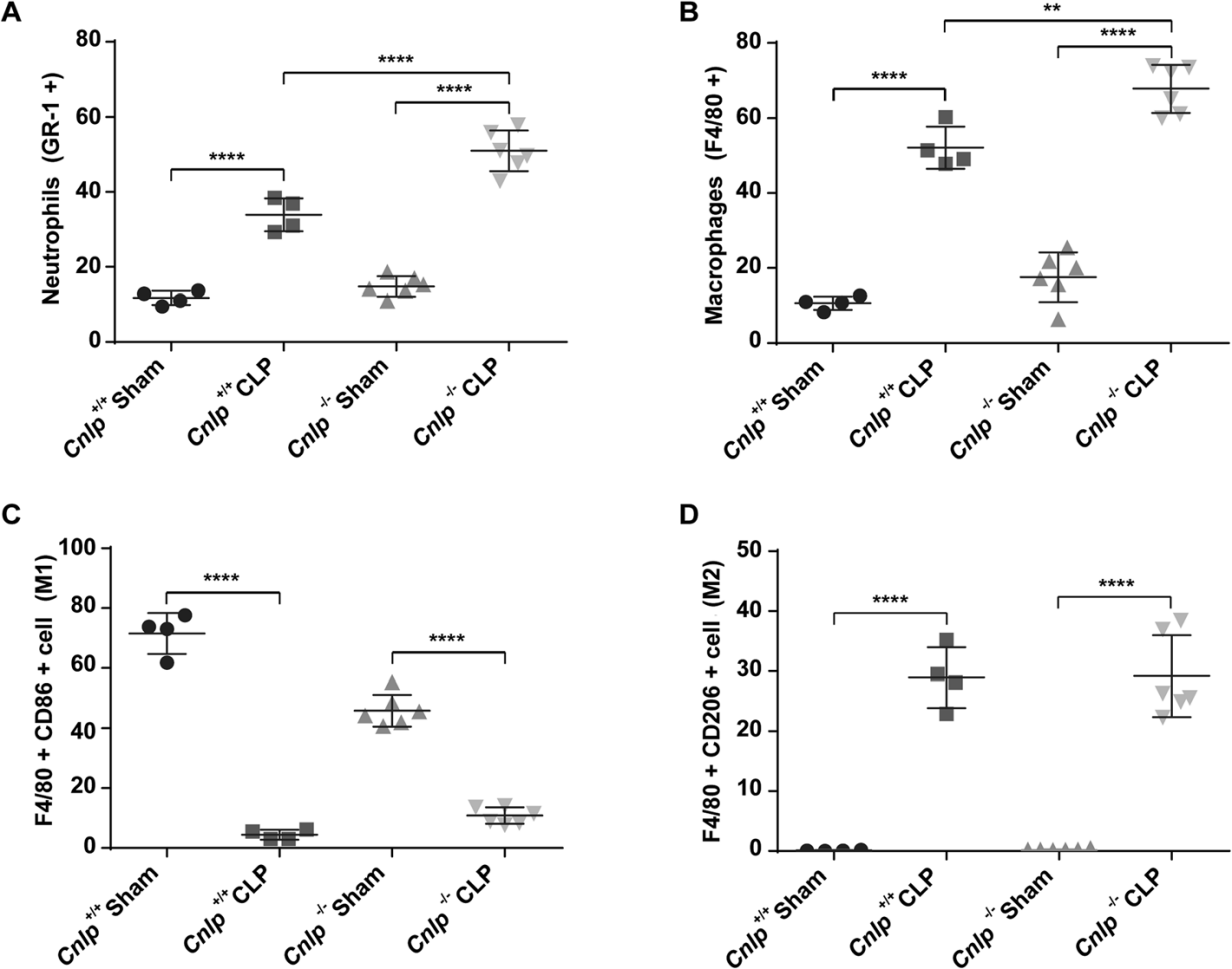
Deletion of endogenous cathelicidin increased neutrophils and macrophages into small intestine. Effects of CLP or sham surgery on the relative proportion of neutrophils and macrophages in small intestine of cathelicidin wild-type (*Cnlp*^+/+^) or knockout (*Cnlp*^−/−^) mice (*n* = 6 per group) at 24 h were determined by flow cytometry. **a** Neutrophils were defined as Ly6G^+^ cells and **b** macrophages as F4/80^+^ cells and **c** M1 macrophages as F4/80^+^ CD86^+^ and **d** M2 macrophages as F4/80^+^ CD206^+^. Error bars denote standard error of the mean. **P < 0.01; ****P < 0.0001

**Fig. 7:**
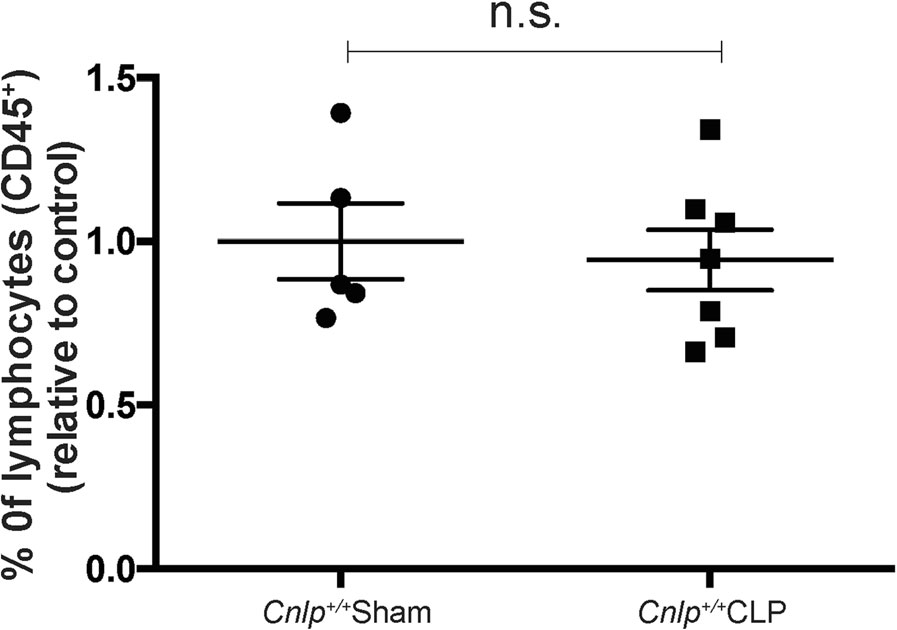
CLP-induced polymicrobial sepsis did not influence the lymphocyte count in the ileum (*n* = 5 for the sham-operated group; *n* = 7 for the CLP group). Flow cytometric analysis of lymphocytes isolated from small intestines of sham-operated or CLP cathelicidin wild type (*Cnlp*^+/+^) was performed. Cells were labeled with anti-mouse CD45^+^ lineage surface markers

Flow cytometry revealed that the number of neutrophils increased by nearly three-fold in wild-type mice 24 h after induction of CLP and depletion of *Cnlp* induced more neutrophil intestinal infiltration as compared with the wild-type mice after CLP (Fig. 6a). Moreover, we observed that CLP significantly increased the number of macrophages in both *Cnlp*^+/+^ and *Cnlp*^−/−^ mice. Compared to wild-type mice at 24 h after CLP, *Cnlp*^−/−^ mice had a higher number of macrophages (Fig. 6b). More specifically, CLP caused a dramatic decline in the percentage of M1 macrophages and depletion of *Cnlp* tended to induce more M1 macrophages compared with the wild-type mice after CLP (Fig. 6c). In contrast, CLP significantly increased the percentage of M2 macrophages, but knockout of *Cnlp* had no effect on the number of M2 macrophages compared with the wild-type mice after CLP (Fig. 6d). In addition to neutrophils and macrophage infiltration, we determined the murine adaptive immunity after CLP. We showed that CLP did not promote the migration of lymphocytes into the ileum at 24 h after CLP (Fig. 7).

### Effect of VD3 on peritonitis-induced polymicrobial sepsis

**Fig. 8:**
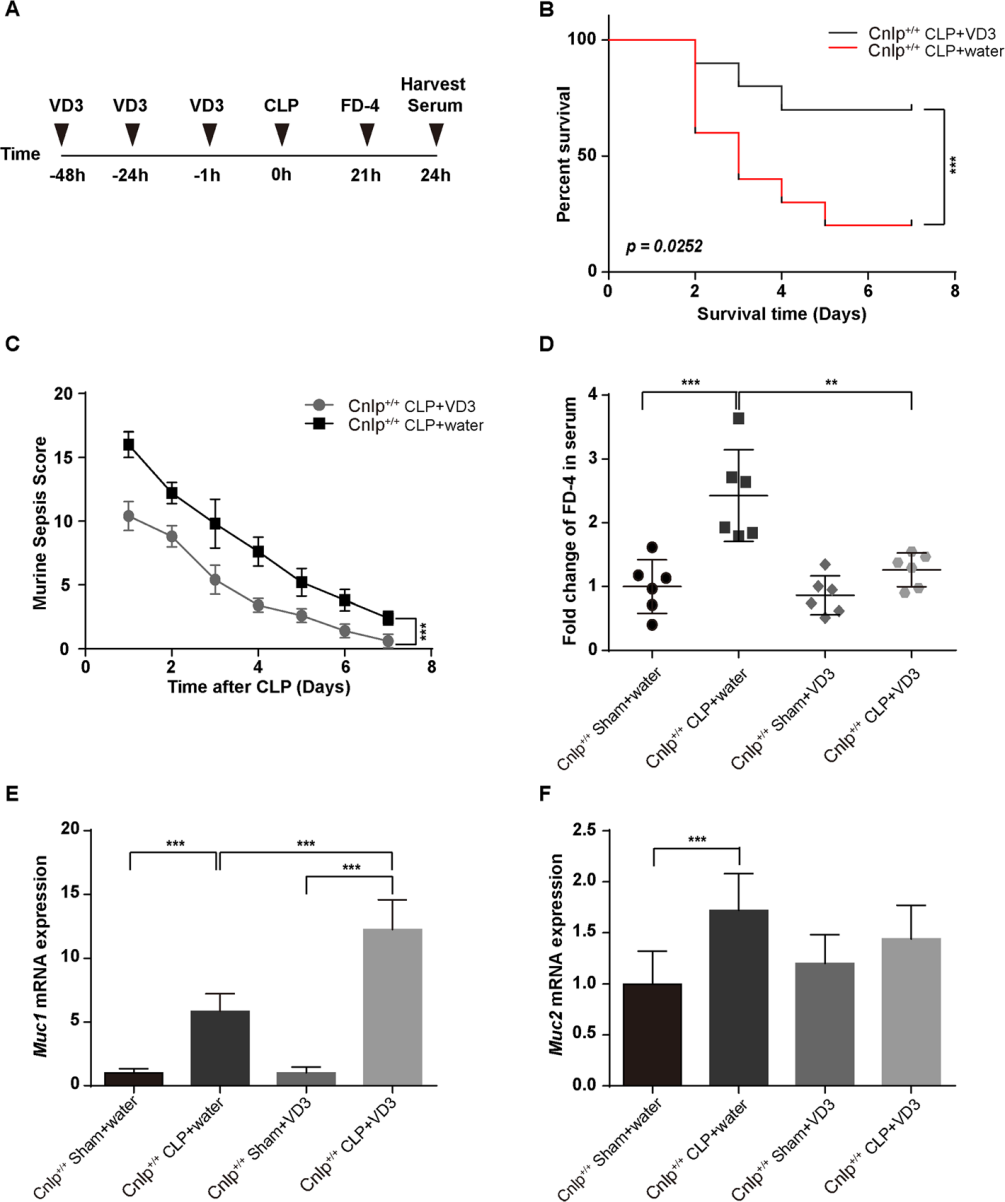
Effect of VD3 on peritonitis-induced polymicrobial sepsis. **a** All mice (*n* = 6 per group) underwent CLP surgery pretreated with water or VD3 by gavage at 48 h, 24 h, and 1 h before CLP. Mice pretreated with VD3 had **b** decreased 7-day mortality (*n* = 10 per group) and **c** significantly lower MSS score (*n* = 10 per group) and **d** lower level of fluorescein dextran entering the bloodstream (*n* = 6 per group). Mucin production increased in mice pretreated with VD3. The expression levels of **e** *Muc1* and **f** *Muc2* were detected in mice pretreated with VD3 compared to mice pretreated with water after CLP-induced sepsis by immunoblotting. Error bars denote standard error of the mean. **P < 0.01; ***P < 0.001; ****P < 0.0001

To determine the significance of VD3 in polymicrobial sepsis, wild-type mice were divided into two groups: water CLP group and VD3 CLP group. All mice underwent CLP pretreated with water or VD3 by gavage at 48, 24, and 1 h before CLP (Fig. 8a). Mice pretreated with VD3 had decreased 7-day mortality (hazard ratio = 0.223, 95% CI 0.060–0.830) (Fig. 8b), significantly lower MSS score (Fig. 8c) and lower levels of fluorescein dextran entering the bloodstream (Fig. 8d).

### Mucin production increased in mice pre-treated with VD3

To further investigate the effect of VD3 on mucin production, we performed real-time quantitative PCR targeting mucin genes *MUC1–2.* Among mice pretreated with VD3, the expression levels of *MUC1* had more than two-fold increased as compared to mice pretreated with water (Fig. 8e). However, the expression levels of *MUC2*id not vary significantly across the experimental groups (Fig. 8e).

### VD3 pre-treatment upregulated ileal expression of cathelicidin in sepsis

**Fig. 9:**
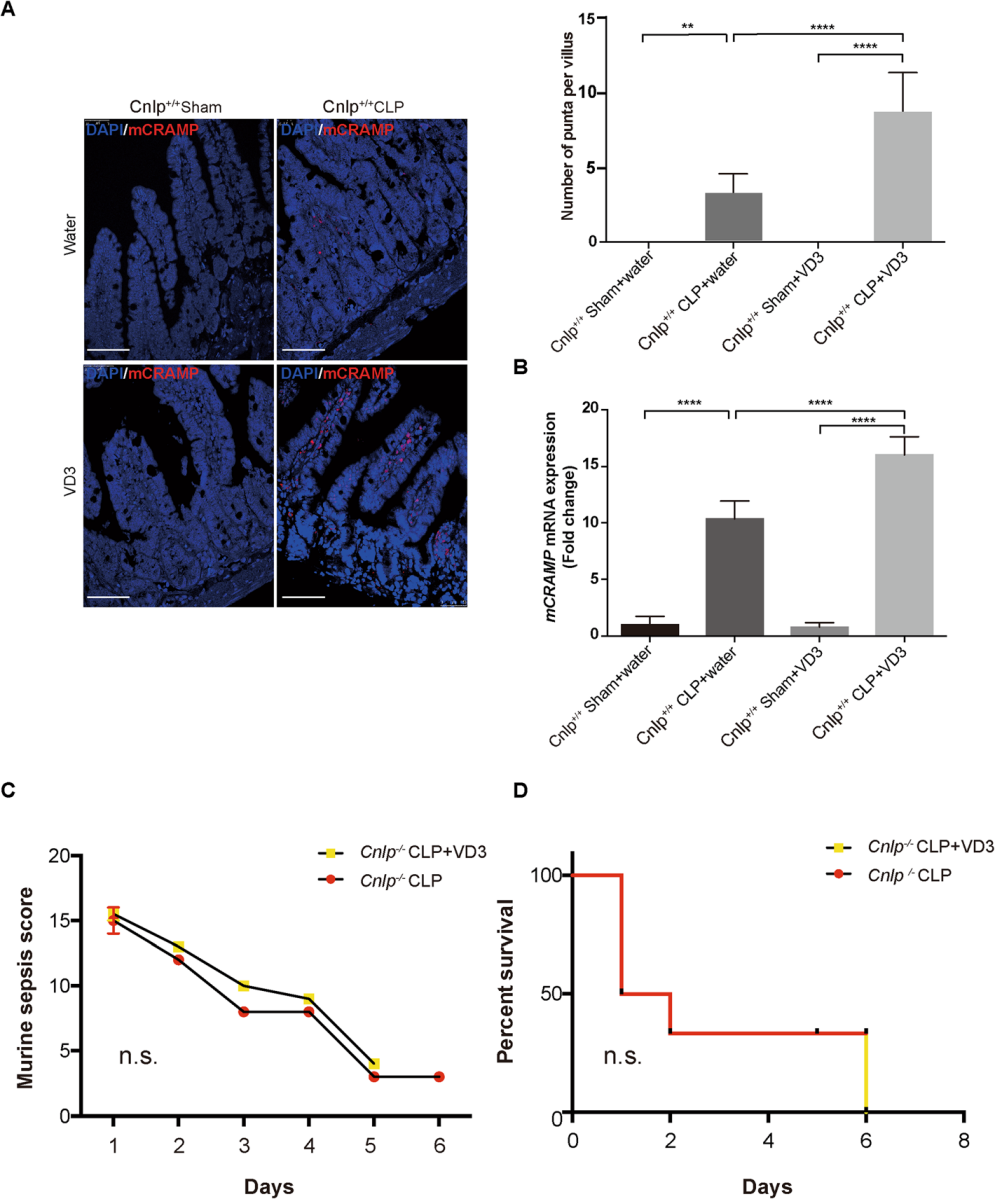
Pretreatment with VD3 up-regulated the ileal expression of cathelicidin in sepsis. Effect of VD3 on expression of mCRAMP in distal ileum of wild-type mice at 24 h after CLP were determined by immunofluorescence and (**a**) real-time quantitative PCR (**b**). *n* = 6 per group, error bars denote standard error of the mean. **c**, **d** Prophylactic efficacy of VD3 required cathelicidin in CLP-induced polymicrobial sepsis. Cathelicidin-knockout mice (*Cnlp*^−/−^) pretreated with water or VD3 by oral gavage at 48 h, 24 h, and 1 h before CLP (*n* = 5 per group). There was no significant difference between water control and VD3 group in terms of **c** 7-day mortality as well as **d** MSS score in *Cnlp*^−/−^ mice. **P < 0.01; ****P < 0.0001

To determine the effect of VD3 on the expression of mCRAMP in distal ileum upon experimental sepsis, immunofluorescent staining for mCRAMP was used. By 24 h after CLP, the number of mCRAMP positive punta per villus increased dramatically compared to mice with sham surgery (Fig. 9a). Moreover, VD3 pretreatment exaggerated the expression of mCRAMP in the distal ileum by more than two-fold (Fig. 9a). At the same time, the result of real-time quantitative PCR revealed a consistent increase in *Cnlp* expression at mRNA level (Fig. 9b). Importantly, the VD3-mediated protective effects could not be observed in CLP mCRAMP-knockout mice (*Cnlp*^−/−^) in terms of murine sepsis score (Fig. 9c) and 7-day mortality (Fig. 9d).

### Effects of inactive and active forms of VD3 on CLP-induced polymicrobial sepsis

**Fig. 10:**
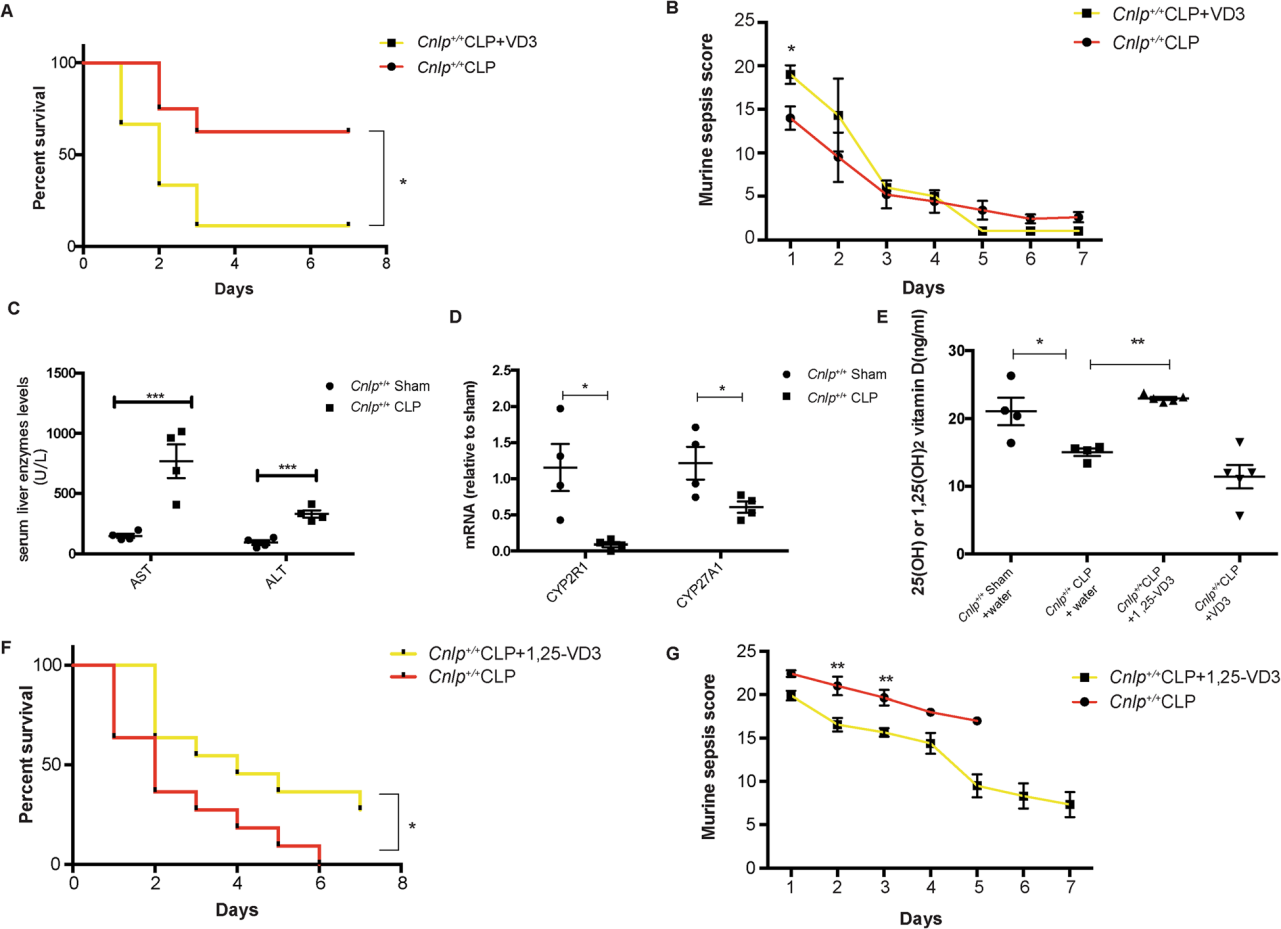
Effects of inactive and active VD3 after the onset of CLP-induced polymicrobial sepsis. **a** All mice underwent CLP surgery and were administered with water or inactive VD3 (50 μg/kg) for one time by oral gavage immediately after CLP (*n* = 8 per group). Mice treated with inactive VD3 even had **a** higher 7-day mortality and **b** higher day-1 MSS score. CLP-induced polymicrobial sepsis resulted in hepatic damage. CLP-operated mice demonstrated increased **c** AST and ALT levels, **d** decreased CYP2R1 and CYP27A1 mRNA levels, and **e** reduced serum VD3 levels (intermediate plus active forms) (*n* = 4–5 per group). For active VD3 treatment, all mice underwent CLP surgery and were administered with water or active VD3 (50 μg/kg) for 7 days by intraperitoneal injection (*n* = 11 per group). Mice treated with active VD3 had better outcomes in terms of **f** 7-day mortality, **g** day 2 and day 3 MSS score as well as **d** higher serum vitamin D3 levels (intermediate plus active forms).*P < 0.05; **P < 0.01; ***P < 0.001

Apart from assessing the prophylactic efficacy of VD3 in polymicrobial sepsis, we further examined the therapeutic potential of VD3 after the onset of sepsis. Results demonstrated that treatment with VD3 after CLP worsened the mortality (Fig. 10a) and the MSS score (Fig. 10b) in the CLP model. Possibly, polymicrobial sepsis resulted in hypoxic hepatitis [24–26]. Under this pathophysiological condition, enzymatic dysfunctions of cytochrome p4502R-1 might fail to hydroxylate the inactive form of vitamin D3 into its intermediate form (i.e., 25-hydroxyvitamin D3) in the liver [27, 28]. Results demonstrated that CLP induced hepatic damage as evidenced by increases in serum ALT and AST levels (Fig. 10c) and suppression of mRNA expression of hepatic cytochrome P450 enzymes CYP2R1 and CYP27A1 (Fig. 10d), both of which are responsible for conversions of cholecalciferol (inactive form of VD3) into 25-hydroxyvitaminD3, eventually resulting in a decrease in serum vitamin D3 level (Fig. 10e). To address this limitation, mice were treated with the active form of VD3 (i.e., 1alpha, 25(OH)_2_VD3; calcitriol) that resulted in better outcomes in terms of 7-day mortality (Fig. 10f), MSS score (Fig. 10g). and serum VD3 levels (Fig. 10e) in the CLP model. Taken together, we found that VD3 and 1alpha, 25(OH)_2_VD3 exerted prophylactic and therapeutic effects in a murine polymicrobial sepsis model, respectively.

## Discussion

Cathelicidin is one of the immunomodulatory proteins involved in the pathogenesis of sepsis [29]. Clinical studies have shown that human cathelicidin was 50% lower in critically ill patients with severe sepsis compared to non-septic patients and was further downregulated in septic shock [30]. Stratification of critically ill patients by different levels of plasma cathelicidin revealed that those with less than 116 ng/mL at admission had four-fold increased risk for 90-day mortality as compared to those with cathelicidin > 238 ng/mL, after controlling for confounders, and also more likely to develop sepsis during the same hospital stay [31]. These suggest that cathelicidin has an important role in sepsis.

In this study, we demonstrated that upon induction of sepsis by CLP in mice, the expression of cathelicidin was increased by four-fold. The increased expression of this peptide was more prominent in the first 4 h upon sepsis induction, indicating that cathelicidin is involved in the acute phase of sepsis. Instead of a sequential occurrence of hyper-inflammation or immunosuppression [32], recent studies suggested a paradigm shift in sepsis pathogenesis in which both processes persist over the course of the disease, leading to persistent inflammation and catabolism syndrome [32]. Given the anti-inflammatory properties of cathelicidin, its gradual decrease at a late stage after CLP in mice may explain why wild-type mice would die at a later stage. The protective role of cathelicidin was also confirmed in survival analysis between *Cnlp* wild-type and knockout group. Consistently, human cathelicidin protects rats against sepsis after bacterial challenge [33] and the increased expression of cathelicidin in adipocytes surrounding the colon limits the release of bacteria from mice with experimental colitis [34]. Nevertheless, contradictory evidence also exists in the literature. Severino et al. reported that wild-type C57BL/6 mice succumbed more rapidly to CLP compared with cathelicidin-deficient mice [35]. The discrepancies between this report and our study might arise from the genetic backgrounds of mice (129/SVJ and C57BL/6, respectively). In this regard, mice of different genetic backgrounds could exhibit divergent antimicrobial activity [36].

Along with the change of cathelicidin expression as revealed by real-time PCR and immunostaining, there were signs of intestinal barrier dysfunction including heightened permeability to fluorescein dextran, reduced mucin production, lowered tight junction protein expression, and increased apoptotic activity. The bacterial load in the blood also became higher after the induction of sepsis. These conditions were further exaggerated in the cathelicidin-knockout mice, whose survival period was significantly shortened after CLP. These confirmed the protective role of cathelicidin in preserving gut barrier function in sepsis.

Mucins are structural components of mucus, which lines the gastrointestinal mucosa, and are important in preventing harmful microbes from entering the bloodstream [37]. The expression of various mucin genes differs upon encountering microbial challenges. Of note, *Muc1* is increased considerably after infection [37], a finding that is in agreement with our observation that *Muc1* and *Muc2* genes were upregulated after induction of experimental sepsis. The magnitude of expression was reduced after knocking out cathelicidin. Although the mechanism of cathelicidin in control of mucin production remains unclear, the administration of exogenous cathelicidin to rats has been shown to increase the thickness of the mucus layer in the intestine [38].

Apoptosis and tight junction alterations are important mechanisms through which intestinal microbes invade the hosts [39]. In our study, we observed higher activity of apoptosis after CLP. This was further exaggerated after knocking out cathelicidin, an antimicrobial peptide that inhibits kidney cell apoptosis by reducing the endoplasmic reticulum stress [40]. The disruption of gut barrier integrity may partially explain the higher bacterial load seen in the cathelicidin-knockout group.

It has been reported that cathelicidin improves septic mice survival by inhibiting pyroptosis of macrophages and preventing exaggerated inflammatory responses [41]. Consistent with this finding, our transcriptome analysis of ileal tissues revealed that expression of inflammatory genes (*Grb2*, *Rela*, *Jun*) were shown as the most popular hub genes (interaction degree large than 20) in the upregulated gene cluster. An increased intestinal inflammatory response has been shown to be associated with gut barrier dysfunction in rodents [42]. Collectively, these suggested that cathelicidin depletion would exaggerate pro-inflammatory response, which was also verified by the KEGG and Reactome pathway analyses. Further mechanistic studies will be needed to determine if cathelicidin controls pro-inflammatory response via *Grb2*, *Rela*, and *Jun*.

It was demonstrated that human cathelicidin synergistically enhanced the endogenous inflammatory mediator interleukin-1β and chemokines such as macrophage chemoattractant proteins in human peripheral blood mononuclear cells [43]. M1 macrophages can rapidly kill pathogens to help the primary host defense, which mainly play a role in pro-inflammation, and M2 macrophages routinely repair and maintain tissue integrity, which serve an anti-inflammatory function [44]. In our study, we observed a dramatic M1-to-M2 shift in the small intestine after CLP and depletion of cathelicidin tended to induce more M1 but not M2 macrophages compared with the wild-type mice after CLP. So 24 h after CLP, the immune state of mice seems immunosuppressive with macrophages polarizing to an M2 phenotype. Given that human cathelicidin directs macrophages differentiation toward proinflammatory macrophages [45], depletion of cathelicidin may lower the pro-inflammation response in the immune environment during CLP. Apart from modulating the function of macrophages, cathelicidin can induce the migration of neutrophils and eosinophils by the formyl-peptide receptor, FPR2 [46]. In our study, CLP induced infiltration of neutrophils into small intestine and depletion of cathelicidin exaggerated neutrophil infiltration compared with the wild-type mice after CLP. Apart from CLP-induced infiltration of macrophages and neutrophils, we examined the infiltration of lymphocytes into the small intestine. Results demonstrated that there is no significant difference between CLP groups and sham groups. In line with our study, two clinical studies reported that there were no significant differences in T cell and B cell populations between septic patients and the corresponding control group [47, 48]. Collectively, these suggested that CLP would induce more infiltration of macrophages and neutrophils into the small intestine. Cathelicidin depletion would exaggerate pro-inflammatory response, which was associated with elevated production of neutrophils and M1 macrophages.

Parekh and colleagues analyzed the patient data of 61 patients with sepsis and utilized the CLP model, demonstrating that sepsis and severe sepsis are associated with vitamin D deficiency, which in turn is associated with more severe sepsis [49]. Accumulating evidence suggests that VD3 exerts protective effects during infections by up-regulating the expression of cathelicidin and beta-defensin 2 in phagocytes and epithelial cells [11]. In our study, we observed that VD3-pretreated mice had better survival after CLP and these mice also recovered faster with a better MSS score. Along with increasing of mucin1 expression, there were signs of upregulation of cathelicidin with VD3 pretreatment as revealed by real-time PCR and immunostaining. The bacterial load in blood became lower in mice after induction of cathelicidin with VD3. These confirmed that VD3 could up-regulate the cathelicidin and protect against sepsis.

Furthermore, we assessed the therapeutic use of the active form and the inactive form of VD3 in our CLP model. We observed that administration of calcitriol (an active form of VD3) but not cholecalciferol (an inactive form of VD3) after the onset of sepsis led to a better survival outcome in CLP mice. In line with recent publications, high-dose VD3 (cholecalciferol, inactive form of VD3) did not improve the survival outcomes of critically ill patients in terms of 90-day mortality [50]. Since hepatic cytochrome P450 (CYPs) play an essential role in the conversion of VD3 into 25-hydroxyVD3 together with additional evidence showing that hepatic CYPs dysfunctions are linked to sepsis [51–53], we further examined the functions of the liver after the onset of sepsis. Our results demonstrated that CLP induced hepatic damage and the associated downregulations of hepatic CYPs at mRNA level, resulting in decreased serum intermediate and active VD3. Fortunately, the administration of calcitriol (an active form of VD3) can bypass hepatic biotransformation of cholecalciferol into 25-hydroxyVD3 mediated by CYP system, directly entering the circulatory system and exerting the beneficial effects. Taken together, we confirmed that the active form of VD3 but not the inactive form of VD3 is a therapeutic drug in our CLP model. Noticeably, the latter worsened 7-day mortality and the associated symptoms in CLP-operated mice, the mechanism of which remains unclear.

This study has potential limitations. First of all, the sample size in our survival analyses is relatively small (*n* = 8–11). Moreover, only male mice were used for behavioral studies, considering the lesser influence of sex hormones on male mice during the estrous cycle. Our results may not be directly applicable to females. Last but not the least, no antibiotics were given to the CLP-operated mice for all experiments, which might undermine the direct extrapolation of our research findings into the clinical settings.

## Conclusion

Cathelicidin is essential in preserving gut barrier function in sepsis. Replenishment of this protein, its induction by VD3 or targeting its immediate downstream molecular moieties may be promising therapies for sepsis to improve clinical outcomes.

## Supplementary information

**Additional file 1: Figure S1.** STEM analysis identified 50 clusters of co-expressed genes which exhibited particular pattern among groups. Only 19 clusters have *p* values less than 0.05 (shown in color). Cluster 8 and 16 were applied for further bioinformatic analysis. Figure S2. Western blots of samples from individual mouse presented in Fig. 3. Figure 3. Western blot of samples from individual mouse presented in Fig. 4.

## Data Availability

The datasets used and/or analyzed during the current study are available from the corresponding author on reasonable request.
