# Supplementary figures and images for: Cathelicidin preserves intestinal barrier function in polymicrobial sepsis
Source: Crit Care. 2020 Feb 10;24:47. doi: 10.1186/s13054-020-2754-5 (PMC7011568; doi:10.1186/s13054-020-2754-5)

Supplementary Figure 1

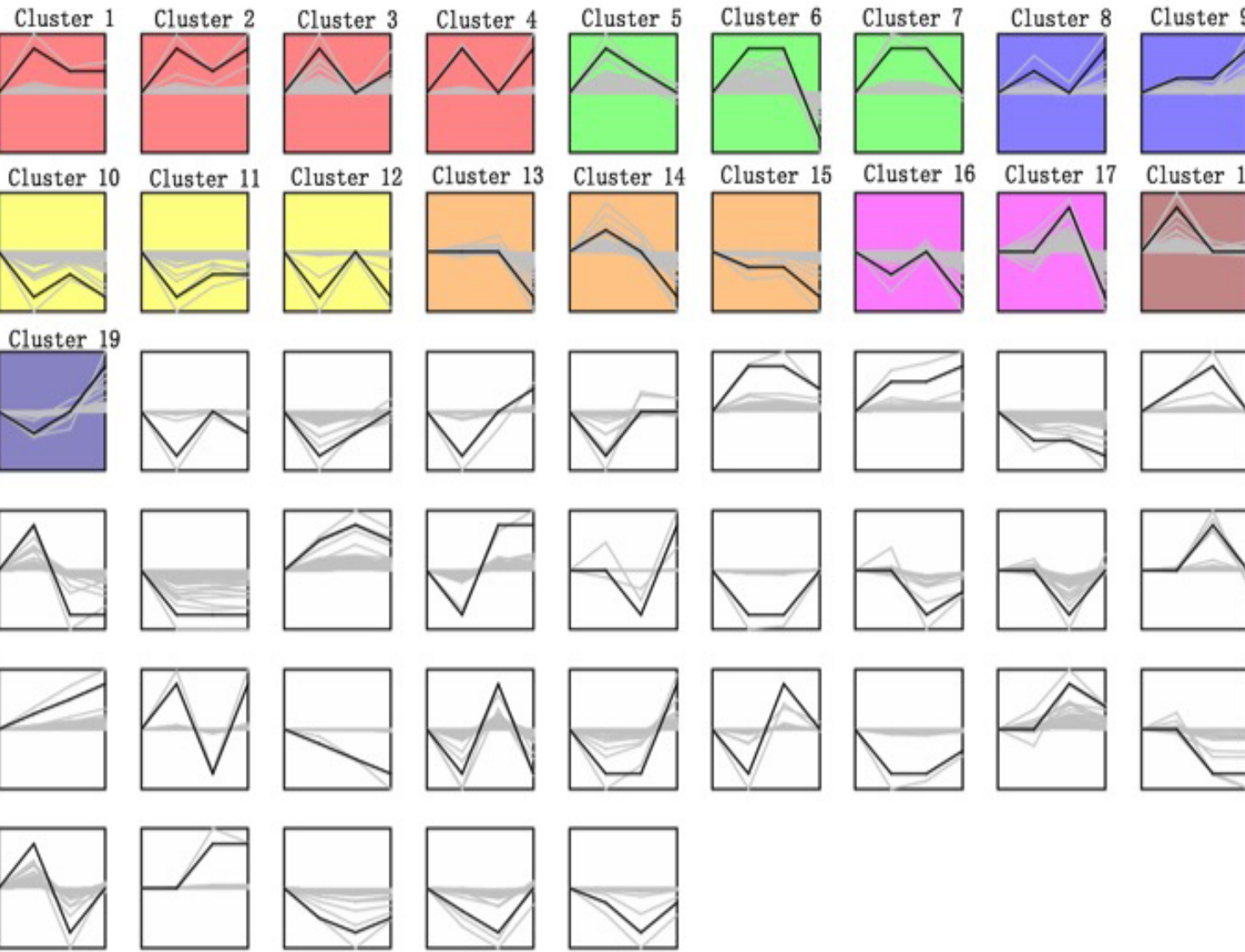

Supplementary Figure 2

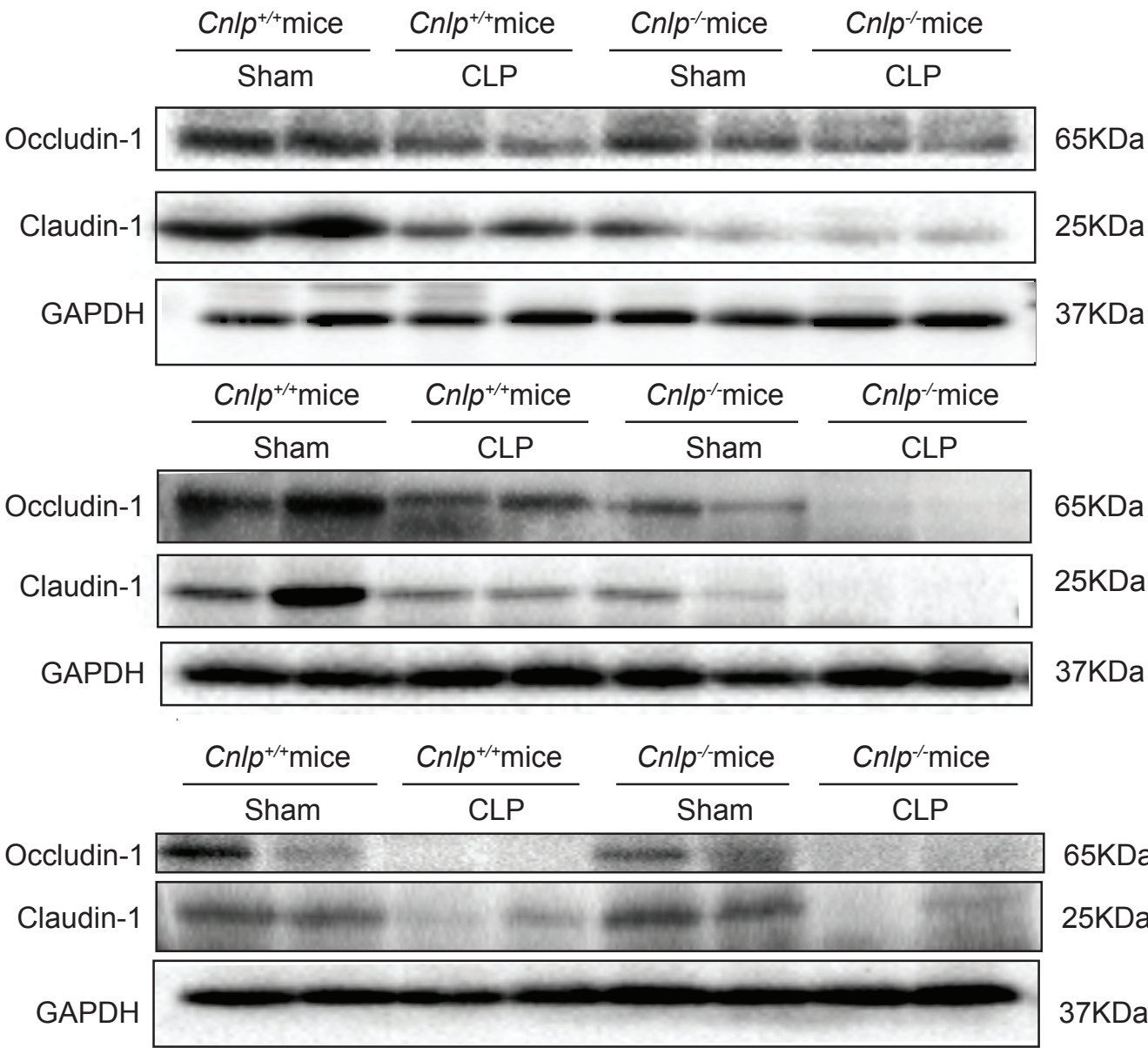

Supplementary Figure 3

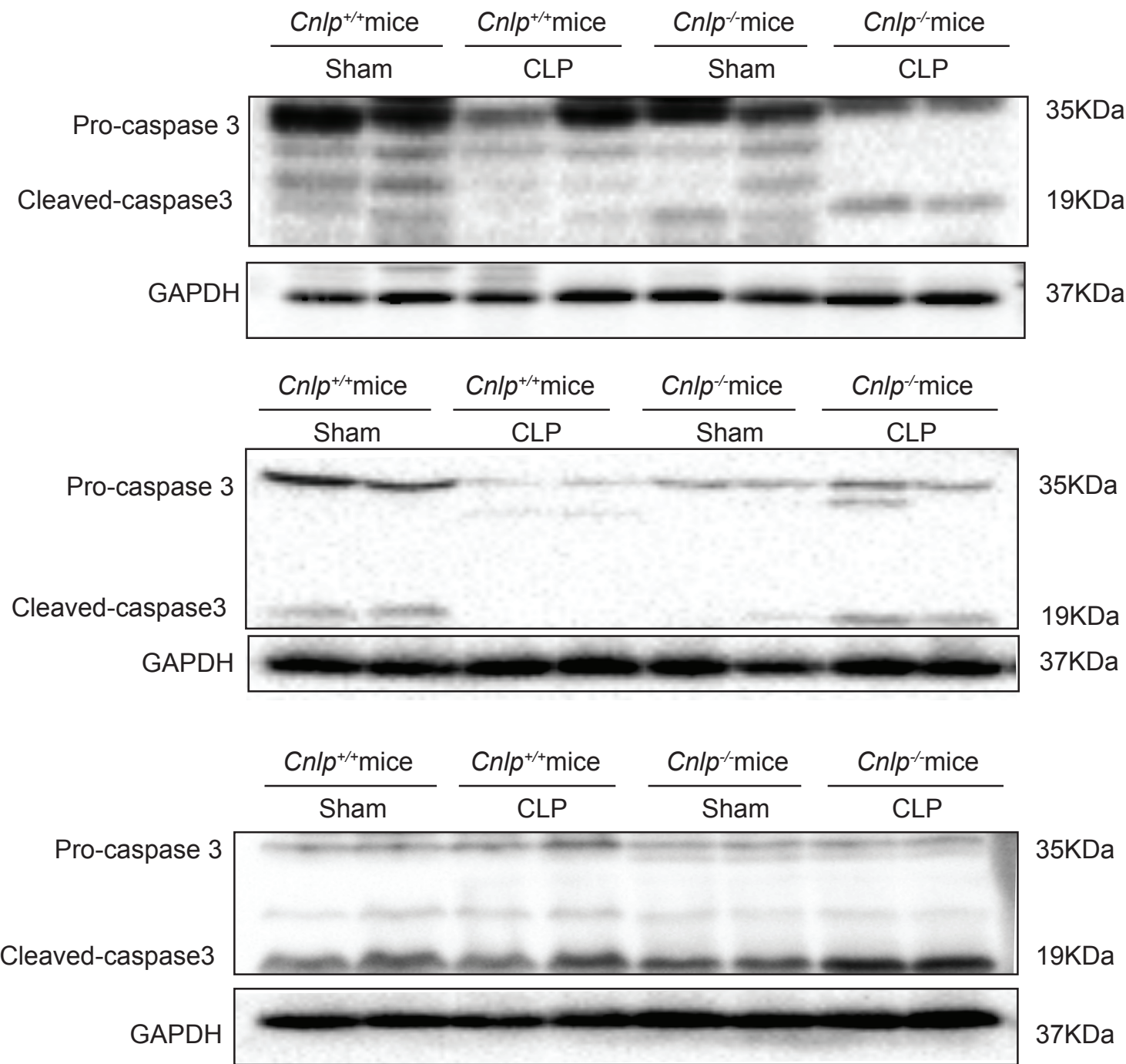

Supplement: Supplementary file 1 — Additional file 1: Figure S1. STEM analysis identified 50 clusters of co-expressed genes which exhibited particular pattern among groups. Only 19 clusters have p values less than 0.05 (shown in color). Cluster 8 and 16 were applied for further bioinformatic analysis. Figure S2. Western blots of samples from individual mouse presented in Fig. 3. Figure 3. Western blot of samples from individual mouse presented in Fig. 4. [file 13054_2020_2754_MOESM1_ESM.pdf]
